# Competing Risks and Multistate Models in Clinical Nephrology Research

**DOI:** 10.1016/j.ekir.2022.08.011

**Published:** 2022-08-27

**Authors:** Katie Ross-Driscoll, Rachel E. Patzer

**Affiliations:** 1 Department of Surgery, Emory University School of Medicine, Atlanta, Georgia, USA; 2 Department of Epidemiology, Emory Rollins School of Public Health, Atlanta, Georgia, USA; 3 Health Services Research Center, Emory University School of Medicine, Atlanta, Georgia, USA; 4 Department of Medicine, Emory University School of Medicine, Atlanta, Georgia, USA

In cohort studies and clinical trials, outcomes are often expressed in terms of time to an event such as time to death, time to treatment, and time to disease incidence. Regression methods that handle time-to-event data are commonly called “survival analysis,” even if death is not the event of interest. Survival analyses allow us to examine not just whether an event happened, but how quickly that event happened.^1^ Survival analysis can also handle patients who are censored (i.e. those who drop out of the study or who do not experience the event of interest by the end of the follow-up period). Instead of treating these patients as having missing event data, survival analysis makes use of all of the follow-up information that is available by including those who experienced a certain amount of time without an event.

Conventional survival analysis, based on Kaplan-Meier methods, has a set of strict assumptions about patients who are censored. The most important of these assumptions is that censoring is not informative (i.e. patients who are censored go on to have the outcome at the same rate as patients who are not censored). This assumption is often not met under real-world conditions in clinical research, where patients experience competing risks to the event of interest.^2^ A competing risk is an event that changes the likelihood that an event will happen, or prevents the event entirely. For example, in a study examining progression to end-stage kidney disease (ESKD) among patients with chronic kidney disease, if the patient died prior to experiencing ESKD, death is a competing event to developing ESKD. Using traditional Kaplan-Meier methods, these patients would be censored at the time of death, and the model would assume that they had the same risk of ESKD as patients who were censored for other reasons, like loss to follow up. However, we know that this assumption is not true. The event of death actually eliminates the likelihood that the patient will develop ESKD. In the presence of this informative censoring, Kaplan-Meier results should be interpreted as the risk of ESKD among chronic kidney disease patients if the probability of death was eliminated. Though in some cases this interpretation may be of interest, for most research questions, we want to know the risk of our event in a real world scenario that includes competing risks.

A variety of survival analysis methods have arisen to handle situations of informative censoring, called competing risk analyses. Competing risks analyses rely on estimating the cause-specific hazard, or the likelihood of experiencing an event from a specific cause in the presence of other causes of that event. One limitation of competing risks analysis, shared by traditional survival analysis, is that it treats all states as final.^3^ This makes sense for some conditions, such as death. However, for other conditions such as diabetic ketoacidosis or graft failure after kidney transplant, the state may actually be temporary. For research questions that involve these “transitional states,” multistate models can be used to approximate a patient’s history of multiple events, not just their time to a single event.^4^ These different methods of survival analysis, their underlying assumptions, and potential research questions are compared in Table 1.

**Table 1:** Approaches, examples, and assumptions of time-to-event analyses in clinical research

Approach	Examples	Assumptions	Example research questions	Limitations
Traditional survival analysis	Kaplan-Meier estimates, Cox proportional hazards regression	• Event of interest can only occur once or only first event is of interest • Censored participants remain same risk of the event as noncensored participants	• How quickly does a single event of interest occur? • How quickly does ESKD develop among patients with CKD?	• Assumes that censoring is not informative and there are no competing risks • Not as realistic for “real world” analyses
Competing risks analysis	Fine & Gray regression, Aalen-Johnston estimators	• Events can only occur once or only the first event is of interest • Once a participant experiences an event from one cause, they are no longer at risk for experiencing the event from another cause	• What is the cumulative probability of the event of interest? • Among patients with CKD, what is the cumulative probability of developing ESKD? What is the cumulative probability of dying before CKD development?	• Treats all states as a final, permanent state • May not be an appropriate approach if the outcome can change
Multistate modeling	Aalen-Johnston estimators, Markov models	• Events can occur multiple times. The entire event history is of interest	• What is the probability that a patient will be experiencing a specific event at a given time? • What is the probability that a patient with acute kidney injury has an eGFR <15 at a given time?	• Difficult to obtain longitudinal data on transitional states or outcomes

CKD, chronic kidney disease; eGFR, estimated glomerular filteration rate; ESKD, end-stage kidney disease.

In this issue’s *KI Reports*, Vanhove *et al.* (unpublished data) demonstrate the importance of the type of survival analysis used in clinical nephrology research. In a study examining kidney transplant outcomes, including graft failure and death, among more than 230,000 adult kidney transplant recipients, the authors demonstrated that competing risk models and multistate models performed better at estimating risks than traditional survival analysis using Kaplan-Meier methods. Traditional survival analysis resulted in age-dependent overestimation of risks of graft failure or death. In other words, among older patients, traditional survival analysis overestimated the risk of poor outcomes, likely because of informative censoring. In addition, the authors demonstrated how multistate models can be used to study a patient’s experience after having graft failure, as compared to competing risk models, which treat graft failure as a permanent state that, once entered, remains constant.

Whereas the findings in Vanhove *et al.* have implications for clinical practice, for example, by quantifying the absolute risk of graft failure among older kidney recipients, the more important implication is for clinical research. The different results given by different types of survival analysis prompt us to consider the assumptions underlying one of our most commonly used methods, and what happens to our results when those assumptions are violated. Clinical researchers must consider these assumptions carefully and select an analysis method for which assumptions are met and the research question of interest can be successfully answered. Data limitations may hamper these efforts. For example, multistate models require detailed longitudinal data collection on a patient’s transition between states. Even if a multistate model is the most appropriate method to answer a question, without complete longitudinal data, researchers are limited to other analytic approaches.

Competing risks and transitional states are far from rare in nephrology. Any report containing time to an event as an outcome should be scrutinized as to which patients are censored and whether censoring might be informative when interpreting outcome measures. Clinical researchers interested in time to event outcomes should be familiar with competing risks and be able to identify when a competing risks approach or multistate approach is the most appropriate to answer their research question.

## Disclosure

Disclosure KRD is supported by the National Center for Advancing Translational Sciences of the National Institutes of Health under Award Number UL1TR002378 and KL2TR002381. The content is solely the responsibility of the authors and does not necessarily represent the official views of the National Institutes of Health.

## References

[1] Clark T.G., Bradburn M.J., Love S.B., Altman D.G. (2003). Survival analysis part I: basic concepts and first analyses. Br J Cancer.

[2] Noordzij M., Leffondré K., van Stralen K.J., Zoccali C., Dekker F.W., Jager K.J. (2013). When do we need competing risks methods for survival analysis in nephrology?. Nephrol Dial Transplant.

[3] Putter H., Fiocco M., Geskus R.B. (2007). Tutorial in biostatistics: competing risks and multi-state models. Stat Med.

[4] Meira-Machado L., de Uña-Álvarez J., Cadarso-Suárez C., Andersen P.K. (2009). Multi-state models for the analysis of time-to-event data. Stat Methods Med Res.

